# Coadministration of rapamycin with a DNA/MVA SIV vaccine improves memory CD8^+^ T cell response

**DOI:** 10.1172/jci.insight.193752

**Published:** 2026-04-23

**Authors:** Shanmugalakshmi Sadagopal, Kasey Stokdyk, Suefen Kwa, Rahul Basu, Sailaja Gangadhara, Rafi Ahmed, Smita S. Iyer, Koichi Araki, Rama Rao Amara

**Affiliations:** 1 Emory Vaccine Center, Emory National Primate Research Center, and; 2 Department of Microbiology and Immunology, Emory University, Atlanta, Georgia, USA.

**Keywords:** AIDS/HIV, Immunology, Infectious disease, AIDS vaccine, Cellular immune response

## Abstract

Inhibiting the mammalian target of rapamycin (mTOR) during acute viral infection generates highly functional memory CD8^+^ T cells. We investigated the effects of inhibiting mTOR by using rapamycin during the effector and contraction phases of the immune response to a DNA prime and Modified Vaccinia Ankara (MVA) boost SIV vaccination in rhesus macaques. Rapamycin administered either during MVA boosts alone (DM_R_) or during both primes and boosts (D_R_M_R_) reduced the contraction of effector CD8^+^ T cells, resulting in higher frequencies of SIV-specific memory CD8^+^ T cells with enhanced quality, as indicated by expression of Bcl2 and CD127. Additionally, rapamycin reduced the frequency of proliferating CCR5^+^CD4^+^ T cells in the blood following the MVA boost. After SIVmac251 infection, rapamycin-treated macaques demonstrated marked expansion of SIV-specific CD8^+^ T cells (reaching up to 50% in blood and 25% in gut). The heightened expansion of SIV-specific CD8^+^ T cells in the DM_R_ group was associated with markedly lower (2-logs compared with unvaccinated and 1-log compared with DM) peak viral load in the gut and set-point viremia, along with improved survival after infection. Thus, inhibiting the mTOR pathway during MVA boosts of a DNA/MVA vaccine enhances vaccine efficacy by improving memory CD4^+^ and CD8^+^ T cell function.

## Abstract 2

In this study, we show that rapamycin treatment during DNA/MVA vaccination enhances CD8 T cell function, reduces SIV targets, and enhances viral control and survival.

## Introduction

Rapamycin was initially discovered as an antifungal agent derived from *Streptomyces hygroscopicus* and was subsequently found to inhibit proliferation of mammalian cells (1, 2). Currently, it is commonly used as an immunosuppressive agent in transplantation patients. Rapamycin specifically inhibits an intracellular serine/threonine kinase, mammalian target of rapamycin (mTOR), which controls cell cycle progression, proliferation, and metabolism in response to cues like growth factors, nutrients, and immune signals (3–5). Several studies have demonstrated the crucial role for mTOR in modulating the immune system, influencing CD8^+^ and CD4^+^ T cells, B cells, dendritic cells, and regulatory T cells (Tregs) (6–8). Rapamycin inhibits the proliferation of most immune cells. However, studies have shown that rapamycin can enhance the magnitude of antigen-specific CD8^+^ T cells and promote the generation of memory CD8^+^ T cells in mice when administered during acute infection or vaccination (7, 9). Additionally, antigen-specific CD8^+^ and CD4^+^ T cells generated in the presence of rapamycin showed superior quality characterized by longevity and capacity for proliferation and protection (9). It is important to note that the dose and timing of rapamycin treatment, relative to the stage of acute infection or vaccination, are critical for achieving beneficial effects on a functional immune response.

Antiviral CD8^+^ T cells with high proliferative capacity play a critical role in controlling viral infections, including lymphocytic choriomeningitis virus (LCMV) (10), SIV (11), and HIV (12). Clinical studies have demonstrated that transplant patients receiving rapamycin had a reduced risk of viral infections, e.g., cytomegalovirus (CMV), compared with patients on other immunosuppressive drugs (13, 14). In pilot nonhuman primate studies, rapamycin treatment enhanced both primary and recall responses after Modified Vaccinia Ankara (MVA) vaccination compared with untreated or tacrolimus-treated controls (15). Rapamycin improved both central (Tcm) and effector (Tem) memory compartments of the vaccinia-specific CD8^+^ T cell response, as well as the frequency of dual cytokine–producing (IFN-γ and TNF-α) effectors.

Rapamycin has also gained interest in the HIV scientific community for its anti-HIV properties. In addition to its potential to enhance virus-specific CD8^+^ T cell responses, rapamycin downregulates expression of CCR5 on CD4^+^ T cells and macrophages, resulting in marked reduction of HIV replication in vitro (16–18). This could be an added advantage for HIV/SIV vaccines because the vaccine-elicited virus-specific CD4^+^ T cells (preferential targets of the virus) (19, 20) may not be infected by the virus if they fail to express the CCR5 viral coreceptor. HIV-infected kidney transplant recipients who were treated with rapamycin had lower frequencies of lymphocytes containing HIV DNA than those treated with other immunosuppressive drugs, suggesting an anti-HIV effect of rapamycin (21).

In this study, we investigated how inhibiting mTOR with rapamycin could enhance the immunogenicity and efficacy of a prime-boost SIV vaccine using DNA primes followed by MVA boosts in rhesus macaques. Specifically, we compared the effects of rapamycin either during MVA boosts or during both DNA primes and MVA boosts. Our results demonstrate important effects of rapamycin on inducing highly functional SIV-specific CD8^+^ T cells and CCR5^lo^ SIV-specific CD4^+^ T cells that contribute to enhanced control of SIVmac251 infection and survival of rapamycin-treated vaccinated macaques.

## Results

### Study design.

**Figure 1:**
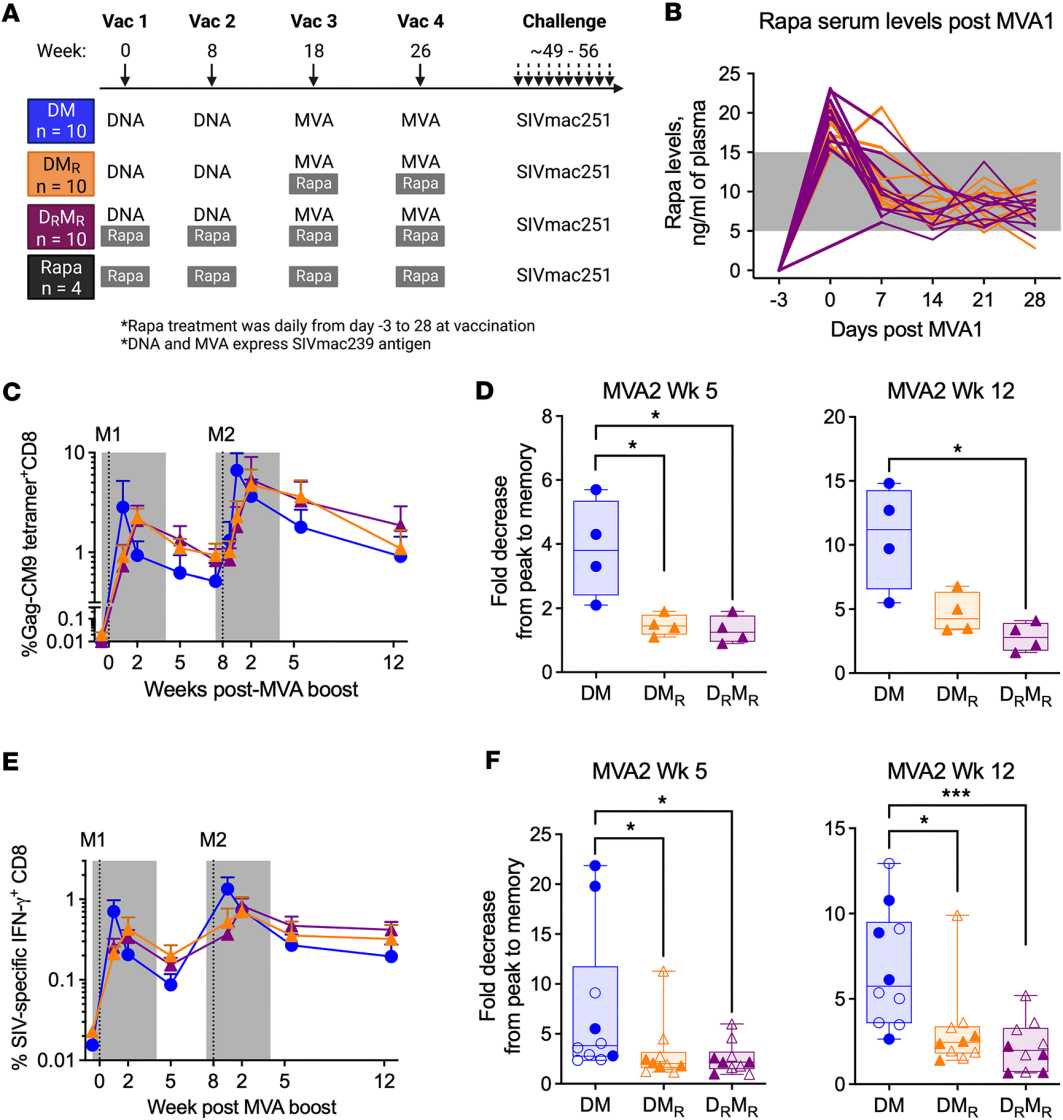
Rapamycin treatment during immunization with DNA/MVA SIV vaccine reduces contraction of Gag-CM9 tetramer^+^ CD8^+^ T cells. (**A**) Macaques were immunized with DNA (D) prime/MVA (M) boost SIV vaccine and treated with rapamycin (Rapa). (**B**) Rapa concentrations in blood for animals in DM_R_ (*n* = 10) and D_R_M_R_ group (*n* = 10). Target concentration range (5–15 ng/mL) is shown in gray. (**C**) Geometric means of frequencies of Gag-CM9–specific tetramer^+^ CD8^+^ T cells measured temporally after MVA boosts in Mamu-A*01 macaques (*n* = 4/group). (**D**) Fold decrease in frequencies of Gag-specific tetramer^+^ cells from peak to memory in the vaccinated groups at weeks 5 and 12 after the second MVA boost. (**E**) Geometric means of frequencies of SIV-specific IFN-γ^+^CD8^+^ T cells measured temporally after MVA boosts. (**F**) Fold decrease in frequencies of IFN-γ on SIV-specific CD8^+^ T cells at week 5 and week 12 after the second MVA immunization (*n* = 10/group). Mamu-A*01 animals are indicated by closed symbols. *P < 0.05; ***P < 0.001 by 1-way ANOVA; 2-way ANOVA multiple comparisons tests was used to compare the fold change between groups.

The primary objective of our study was to determine whether inhibiting mTOR during the T cell expansion and effector-to-memory transition phases following vaccination enhances the CD8^+^ and CD4^+^ T cell memory induced by vaccination in the setting of a DNA prime, MVA boost (DM) SIV vaccine platform. To this end, male rhesus macaques were treated with rapamycin from day –3 to day +28, either during MVA boosts alone (DM_R_; *n* = 10) or during both the DNA primes and MVA boosts (D_R_M_R_; *n* = 10) (Figure 1A). Vaccinated animals not treated with rapamycin (DM; *n* = 10) served as vaccine-only controls, and unvaccinated animals treated with rapamycin served as an additional set of rapamycin-only controls (*n* = 4). To achieve concentrations of 5–15 ng rapamycin/mL blood (as generally seen in transplant recipients, ref. 22), animals were administered a loading dose of 0.03 mg/kg rapamycin subcutaneously on day –3. Subsequently, drug levels were monitored weekly, and dosage was adjusted resulting in trough levels of 5–15 ng/mL throughout the 32-day treatment period (Figure 1B). We monitored blood chemistry, liver enzymes, kidney enzymes, and hematology following the second MVA. Rapamycin treatment did not significantly alter any of these (Supplemental Figure 1; supplemental material available online with this article; https://doi.org/10.1172/jci.insight.193752DS1). Starting on week 49, we administered intrarectal challenges of SIVmac251 weekly to all animals, including unvaccinated controls (*n* = 10), to evaluate protection.

### Rapamycin treatment reduces contraction of vaccine-induced CD8^+^ T cells.

To determine whether rapamycin during vaccination regulated the magnitude of the CD8^+^ T cell response, we assessed frequencies of Gag-CM9–specific tetramer^+^ cells in Mamu A*01^+^ animals (*n* = 4/vaccine group; Figure 1, C and D, and Supplemental Figure 2A) and frequencies of IFN-γ^+^ Gag- and Env-specific CD8^+^ T cells by intracellular cytokine staining (ICS) at peak effector and memory time points after MVA boosts (Figure 1, E and F, Supplemental Figure 2B). Consistent with rapamycin-induced regulation of cell proliferation, CD8^+^ T cell response peaked at week 2 with rapamycin treatment relative to week 1 in vaccinated macaques without rapamycin (Figure 1, C and E). Strikingly, after MVA2, the contraction of Gag-CM9 tetramer^+^ cells from peak to memory was lower in rapamycin groups (1.3-fold at week 5 and 2.8-fold at week 12 in D_R_M_R_, and 1.5-fold at week 5 and 4.7-fold at week 12 in DM_R_) relative to DM (3.8-fold at week 5 and 10.7-fold at week 12) (Figure 1D). Correspondingly, SIV-specific IFN-γ^+^CD8^+^ T cell responses as assessed by ICS in all animals irrespective of Mamu A*01 status showed a similar expansion and contraction kinetics of SIV Gag- and Env-specific CD8^+^ T cells in blood after MVA1 and MVA2 (Figure 1E and Supplemental Figure 1B). Again, the magnitude of peak IFN-γ^+^CD8^+^ T cells was not altered by rapamycin, but the contraction of effector CD8^+^ T cells showed a significant decrease in the group that was treated with rapamycin at the DNA prime and MVA boost, which resulted in a trend for higher magnitude of memory IFN-γ^+^CD8^+^ responses (Figure 1, E and F).

### Rapamycin treatment enhances development of a central memory phenotype with enhanced survival capacity on SIV-specific CD8^+^ T cells.

**Figure 2:**
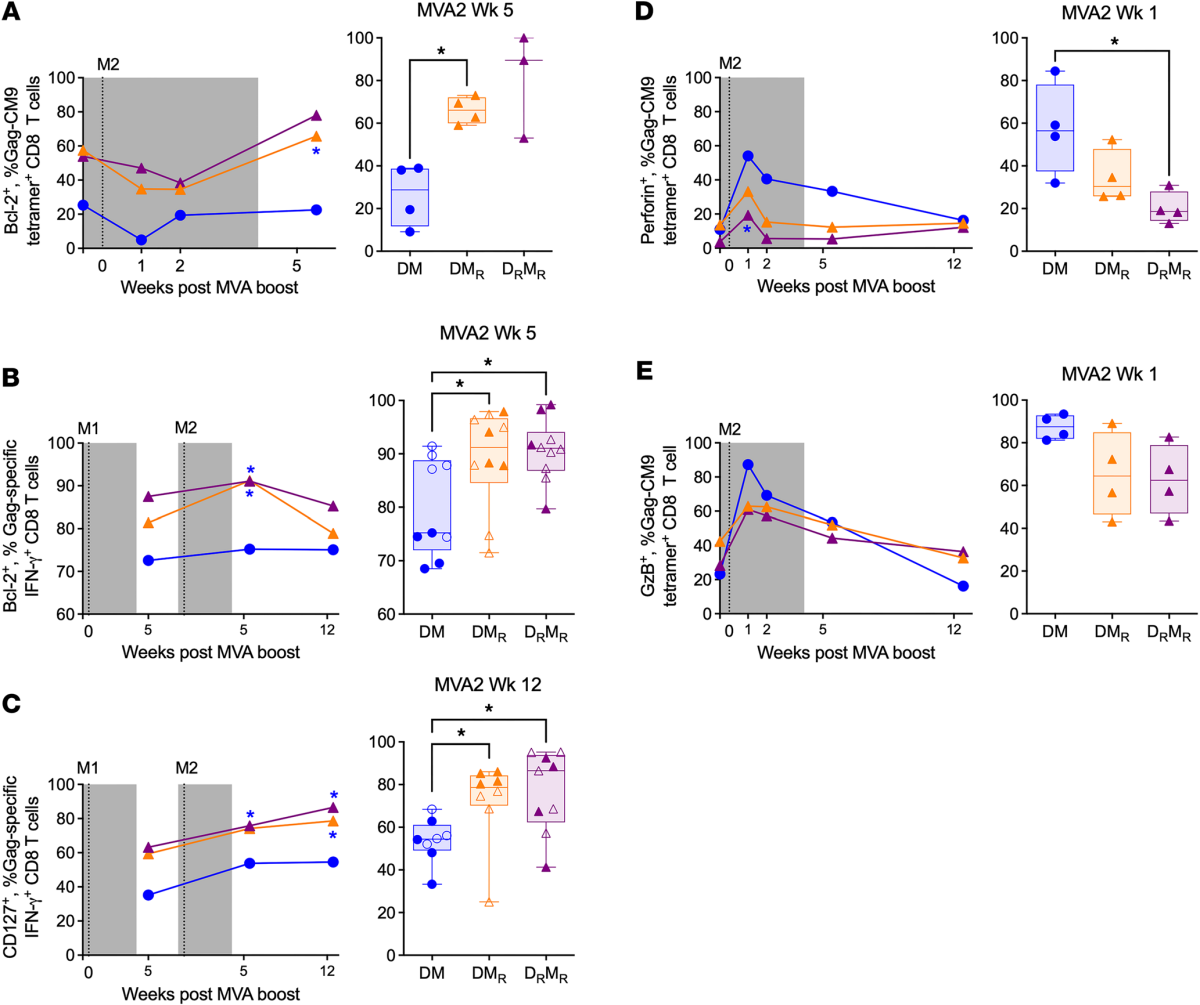
Rapamycin treatment enhances development of central memory phenotype with enhanced survival capacity on SIV-specific CD8^+^ T cells. (**A**) Geometric means of Bcl2^+^ cells on Gag-CM9 tetramer^+^ CD8^+^ T cells measured temporally after the second MVA boost in Mamu-A*01 macaques (*n* = 4/group) and individual data at MVA2 week 5. (**B**) Median frequencies of Bcl2 on Gag-stimulated IFN-γ^+^CD8^+^ T cells measured temporally after MVA immunizations in PBMCs of vaccinated animals (*n* = 10/group) and individual data at MVA2 week 5. (**C**)Median frequencies of CD127 on Gag-stimulated IFN-γ^+^CD8^+^ T cells measured temporally after MVA immunizations in PBMCs of vaccinated animals and individual data at MVA2 week 12. (**D**) Geometric means of perforin on Gag-CM9–specific tetramer^+^ cells measured temporally after MVA immunizations in Mamu-A*01 macaques (*n* = 4/group) and individual data at MVA2 week 1. (**E**) Geometric means of granzyme B (GzB^+^) on Gag-CM9–specific tetramer^+^ cells measured temporally after MVA immunizations in Mamu-A*01 macaques (*n* = 4/group) and individual data at MVA2 week 1. Mamu-A*01 animals are indicated by closed symbols. *P < 0.05 by 1-way ANOVA; 2-way ANOVA multiple comparisons tests was used to compare the fold change between groups.

Since rapamycin improves the longevity of virus-induced memory CD8^+^ T cells (9), we next sought to understand phenotypic features of vaccine-induced CD8^+^ T cells. To this end, we quantified expression of Bcl2 (marker for increased survival capacity), CCR7 (marker for lymph node homing potential and Tcm cells), and CD127 (marker for long-lived memory T cells) on vaccine-induced CD8^+^ T cells. Consistent with the enhancement of CD8^+^ T cell memory with rapamycin, levels of Bcl2 on Gag-CM9 tetramer^+^ cells and IFN-γ^+^ SIV-specific CD8^+^ T cells (Figure 2, A and B, and Supplemental Figure 3A), and CD127 on IFN-γ^+^ SIV-specific CD8^+^ T cells after MVA boost was higher with rapamycin (Figure 2C).

We observed a trend for increase in CCR7 expression on tetramer^+^ CD8^+^ T cells in rapamycin-treated macaques (Supplemental Figure 3, A and B). In contrast to their expression on vaccine-induced CD8^+^ T cells, Bcl2 and CD127 expression on total CD8^+^ T cells was similar across vaccine groups, indicating the effects of rapamycin were specific to vaccine-induced CD8^+^ T cells (Supplemental Figure 3D). Thus, rapamycin enhanced phenotypic features of Tcm cells. Correspondingly, assessment of cytolytic markers perforin and granzyme B (markers for cytotoxic effector T cells) revealed a trend for lower expression on Gag-CM9 tetramer^+^ cells in rapamycin-treated macaques (Figure 2, D and E).

### Viral control and survival was enhanced in rapamycin-treated macaques after intrarectal SIVmac251 challenge.

**Figure 3:**
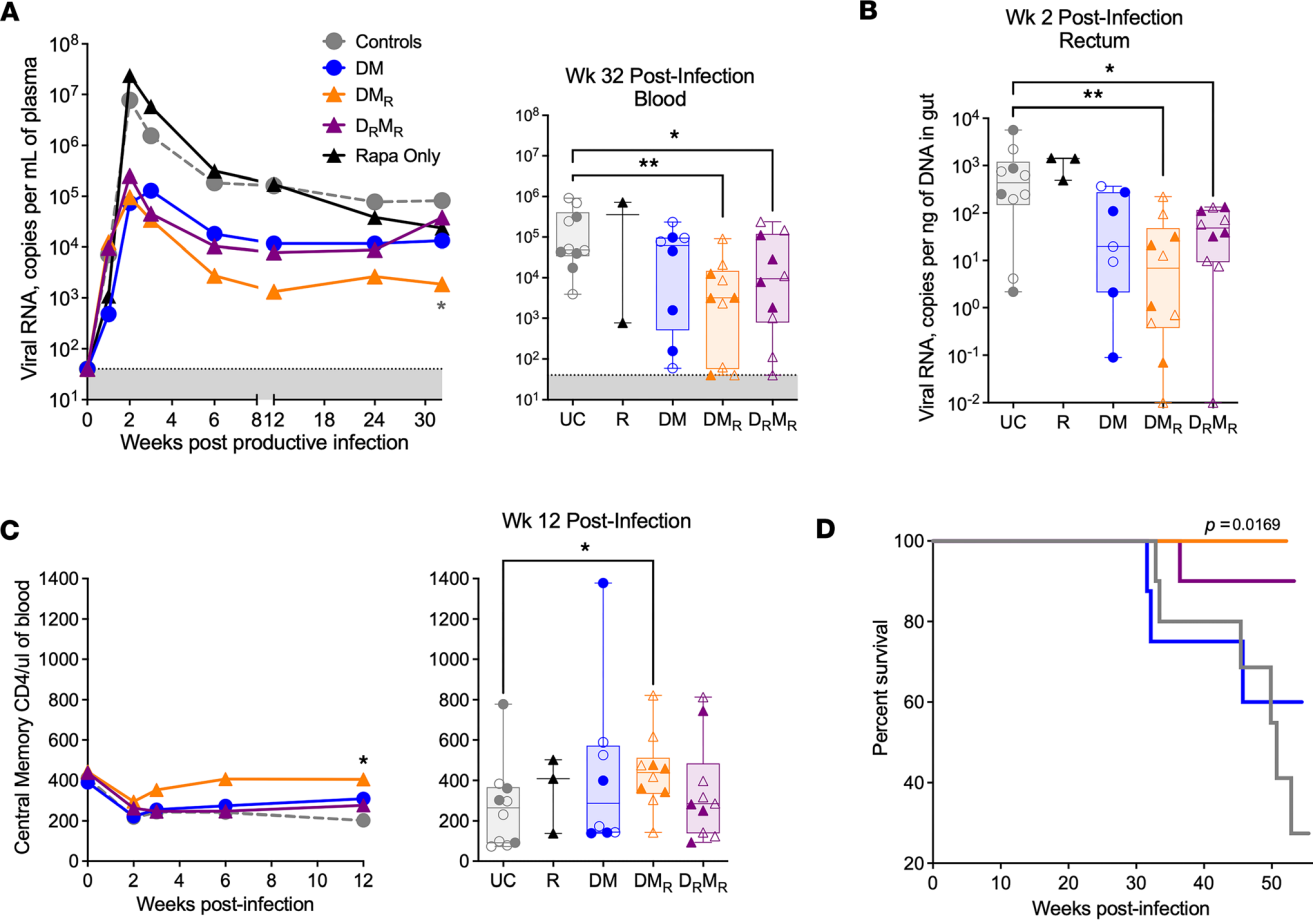
Better viral control and survival in rapamycin-treated macaques. Macaques were challenged intrarectally with SIV_mac251_ at around 29 weeks after final vaccination. (**A**) Left: Geometric means of SIV RNA copies/mL of plasma measured temporally (*n* = 10/group). Right: SIV RNA copies/mL of plasma shown individually at 32 weeks after infection. (**B**) SIV RNA copies in the rectum at week 2 after infection. (**C**) Left: Geometric means of absolute central memory CD4^+^ T cell counts measured temporally (*n* = 10/group). Right: Absolute central memory CD4^+^ T cell counts shown individually at 12 weeks after infection. (**D**) Kaplan-Meier survival curve after infection. Mamu-A*01 animals are indicated by closed symbols. *P < 0.05, **P < 0.01 by 1-way ANOVA; 2-way ANOVA multiple comparisons tests were used to compare viral loads and CD4^+^ central memory counts between groups at various time points, except for CCR5^+^ cells among Ki67^+^ CD4^+^ T cells, for which we used an unpaired, non-parametric Mann-Whitney test. Log-rank (Mantel-Cox) test was used for the survival curve.

To assess whether the enhanced central memory phenotype of Gag-specific CD8^+^ T cells induced by rapamycin resulted in improved viral control, macaques were challenged intrarectally with SIVmac251 (weekly, repeat-dose). While no differences in acquisition rates were observed (Supplemental Figure 4), rapamycin-treated groups demonstrated enhanced viral control from 6–32 weeks after infection, with a 1.6–2.4 log better viral control in DM_R_ relative to unvaccinated controls and 0.6–0.9 log viral control compared with the DM group (Figure 3A and Supplemental Figure 5). At 32 weeks after infection, enhanced viral control was observed in both rapamycin-treated vaccinated groups relative to unvaccinated controls (Figure 3A). Examination of viral loads in the gut at week 2 after infection demonstrated significantly lower vRNA copies in vaccinated macaques, with the DM_R_ group eliciting the most robust viral control in this compartment (Figure 3B). However, viral load was not significantly lower than the DM group in either the blood or the rectum, suggesting that the addition of rapamycin does not significantly promote viral control. To delineate whether enhanced viral control resulted in preservation of CD4^+^ T cells, we assessed the CD4^+^ Tcm cells in blood and observed significantly higher Tcm in the DM_R_ group compared with unvaccinated controls (*P* = 0.02, Figure 3C). In line with enhanced CD4^+^ T cell preservation and viral control, rapamycin-treated macaques demonstrated enhanced survival relative to untreated (control) or vaccinated, untreated (DM) macaques, with significantly higher survival rates in the DM_R_ group compared with DM (*P* = 0.03) or unvaccinated controls (*P* = 0.02) assessed between weeks 45 and 55 after infection (Figure 3D). Thus, rapamycin treatment during DNA/MVA vaccination resulted in a higher survival rate than both controls and vaccinated, untreated animals, and enhanced set-point viral control and preservation of CD4^+^ Tcm counts after infection compared with controls.

### Rapamycin reduces expression of CCR5 on vaccine-specific CD4^+^ T cells.

**Figure 4:**
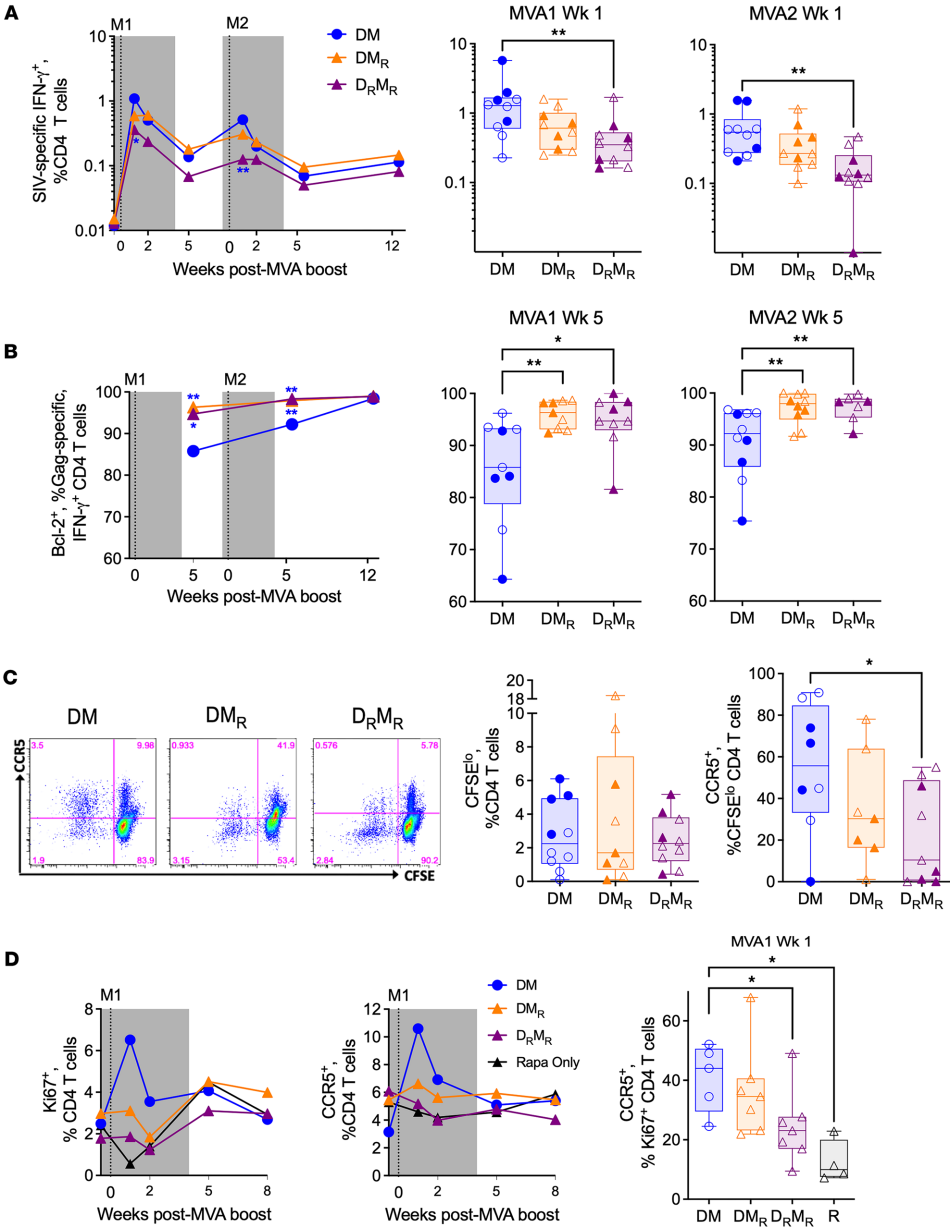
Rapamycin reduces expression of CCR5 on vaccine-specific CD4^+^ T cells. (**A**) Geometric means of frequencies of SIV-specific IFN-γ^+^CD4^+^ T cells measured temporally after MVA immunization in stimulated PBMCs after vaccination. Individual data points at MVA1 week 1 and MVA2 week 1 (*n* = 10/group). (**B**) Median frequencies of Bcl-2 on SIV-Gag–stimulated CD4^+^ T cells measured temporally after MVA immunization in stimulated PBMCs of immunized animals. Individual data at MVA1 week 5 and MVA2 week 5 (*n* = 10/group). (**C**) Representative flow plot of CCR5 and CFSE on SIV-Gag–stimulated CD4^+^ T cells from MVA2 week 12 time points. Frequencies of proliferating (CFSE^lo^) CD4^+^ T cells, and frequencies of CCR5^+^ cells among proliferating (CFSE^lo^) CD4^+^ T cells upon stimulation with pools of Gag and Env peptides. (**D**) Geometric means of Ki67^+^ and CCR5^+^ cells among total CD4^+^ T cells measured temporally after MVA immunizations. Individual frequencies of CCR5^+^ cells among Ki67^+^CD4^+^ T cells at MVA week 1. Mamu-A*01 animals are indicated by closed symbols. *P < 0.05; **P < 0.01 by 1-way ANOVA; 2-way ANOVA multiple comparisons tests were used to compare marker-positive frequencies between groups at various time points, except for CCR5^+^ cells among Ki67^+^CD4^+^ T cells, for which we used an unpaired, non-parametric Mann-Whitney test. For BCl2 data shown in **B**, we did not have data for 2 or 3 animals. For the CFSE proliferation data shown in **C**, we did not perform the assay on 1 animal in the DM_R_ group due to insufficient cells. For the CCR5 expression on proliferating cells in **C**, we considered only samples in which proliferation was at least 1%. This excluded 6 animals (2 in DM, 3 in DM_R_, and 1 in D_R_M_R_).

To better understand the mechanistic basis of CD4^+^ T cell preservation after infection, we assessed CD4^+^ T cell responses induced after vaccination. Rapamycin treatment during DNA/MVA vaccination decreased peak frequencies (week 1 after each MVA boost) of IFN-γ–producing SIV-specific CD4^+^ T cells in the D_R_M_R_ group compared with the DM group (Figure 4A). This decrease was not significant in the DM_R_ group, which received rapamycin only during the MVA. However, by the memory time point (12 weeks after the second MVA), no significant differences persisted between the groups the in frequency of IFN-γ–producing SIV-specific CD4^+^ T cells. To assess the quality of SIV-specific CD4^+^ T cells, we quantified the expression of Bcl2. Nearly 100% of IFN-γ^+^ SIV-specific CD4^+^ T cells in rapamycin-treated macaques expressed Bcl2 at the 5-week memory time point after MVA boosts, a significant increase compared with untreated macaques (Figure 4B).

To determine whether enhanced preservation of CD4^+^ T cells after infection in rapamycin-treated groups was attributable to a decrease in CCR5, we assessed CCR5 reexpression on vaccine-specific CD4^+^ T cells responding to restimulation using an in vitro CSFE-based proliferation assay (Figure 4C). While rapamycin treatment did not alter ex vivo proliferative capacity of CD4^+^ or CD8^+^ T cells (data not shown for CD8^+^ T cells), reexpression of CCR5 on proliferating SIV-specific CD4^+^ T cells (Figure 4C) and Ki67^+^ cells (Figure 4D) was significantly reduced in the D_R_M_R_ group compared with the DM group. These data indicate that rapamycin treatment resulted in decreased frequencies of potential virus target cells while preserving the SIV-specific CD4^+^ T cell help during infection.

### Rapamycin during vaccination enhances magnitude and quality of postinfection CD8^+^ and CD4^+^ T cell responses.

**Figure 5:**
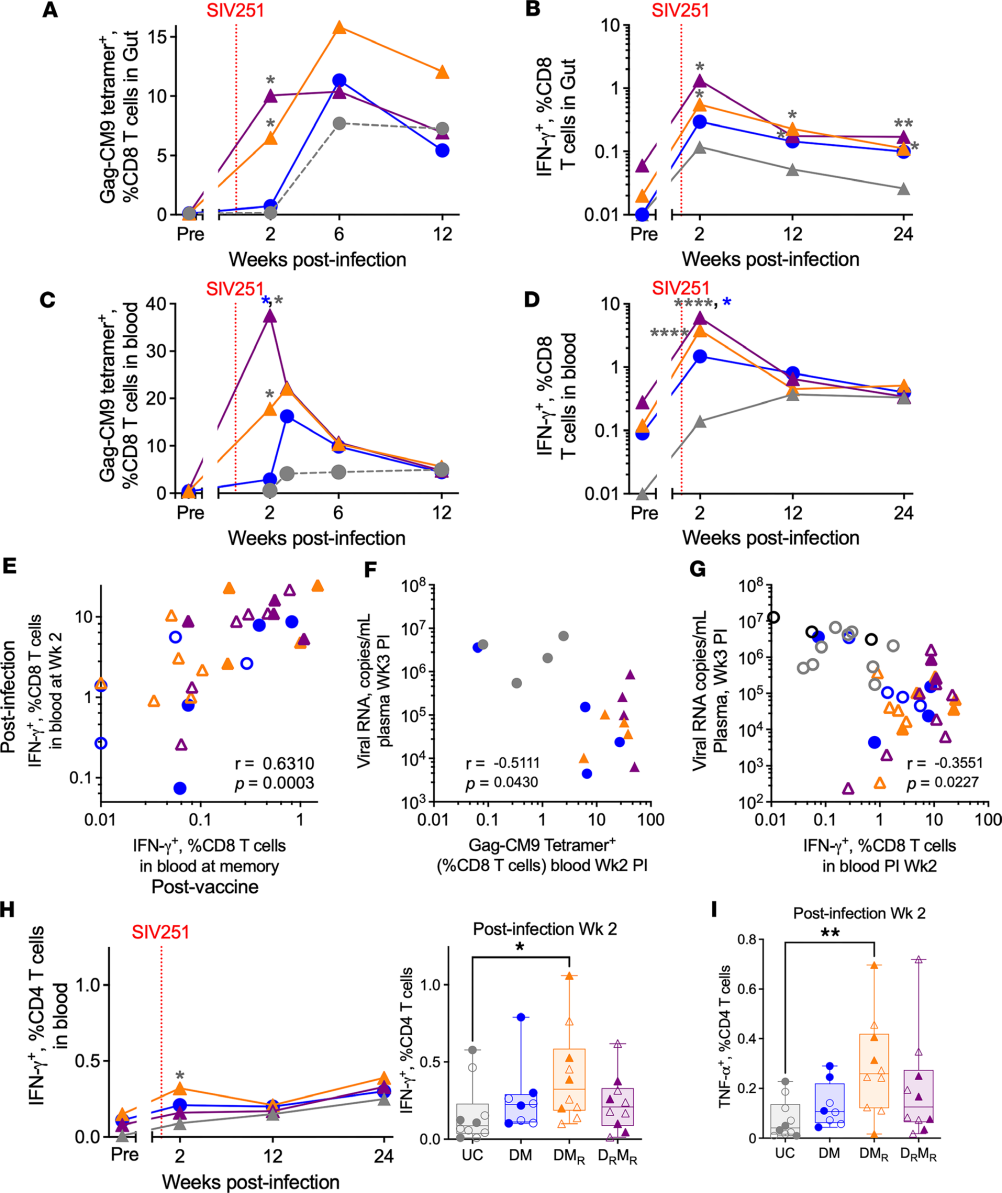
Enhanced magnitude and quality of postinfection recall CD8^+^ and CD4^+^ T cell responses in rapamycin-treated macaques. (**A**) Geometric means of frequencies of Gag-CM9–specific tetramer^+^ cells among total CD8^+^ T cells measured temporally after infection in the gut of Mamu-A*01 macaques (*n* = 4/group). (**B**) Geometric means of SIV-specific IFN-γ^+^ cells among total CD8^+^ T cells measured temporally after infection in the gut (*n* = 10/group). (**C**) Geometric means of frequencies of Gag-CM9–specific tetramer^+^ cells among total CD8^+^ T cells measured temporally after infection in the blood of Mamu-A*01 macaques (*n* = 4/group). (**D**) Geometric means of SIV-specific IFN-γ^+^ cells among total CD8^+^ T cells measured temporally after infection in the blood (*n* = 10/group). (**E**) Correlation of frequencies of postinfection SIV-specific IFN-γ^+^CD8^+^ T cells with postvaccination memory SIV-specific IFN-γ^+^CD8^+^ T cells in blood. (**F**) Correlation of frequencies of postinfection (PI) Gag-CM9 tetramer^+^ CD8^+^ T cells in blood on week 2 after infection with plasma viral load at week 3 after infection. (**G**) Correlation of SIV-specific IFN-γ^+^CD8^+^ T cells in blood with plasma viral load at week 3 after infection. (**H**) Left: Geometric means of SIV-specific IFN-γ^+^ cells among total CD4^+^ T cells measured temporally after infection in blood (*n* = 10/group). Right: Frequency of IFN-γ^+^ cells among total CD4^+^ T cells shown individually at postinfection week 2 (*n* = 10/group). (**I**) Frequency of TNF-α^+^ cells among total CD4^+^ T cells shown individually at postinfection week 2 (*n* = 10/group). Mamu-A*01 animals are indicated by closed symbols. *P < 0.05, **P < 0.01 by 1-way ANOVA; 2-way ANOVA multiple comparisons were used to compare cytokine-positive frequencies between groups.

Based on enhanced viral control, we sought to gain an understanding of postinfection T cell responses. To this end, frequencies of SIV-specific CD8^+^ T cells in gut and blood were quantitated by tetramer staining or ICS for IFN-γ and TNF-α temporally after infection. Peak CD8^+^ T cell recall responses were significantly higher in both the rapamycin-treated groups compared with the DM group in gut (Figures 5, A and B) and blood (Figure 5, C and D). Peak postinfection IFN-γ responses in blood were highly correlated with postvaccine IFN-γ frequencies in blood at memory (Figure 5E), indicating potency of rapamycin-induced vaccine-specific CD8^+^ memory T cells. Moreover, the frequency of Gag-CM9 tetramer–specific and Gag-specific IFN-γ^+^CD8^+^ T cells in blood were inversely correlated with viral load at week 3 after infection, suggesting the contribution of CD8^+^ T cells in viral control (Figure 5, F and G). We also measured SIV-specific CD4^+^ T cells in blood after infection. At 2 weeks after infection, frequencies of CD4^+^IFN-γ^+^ and CD4^+^TNF-α^+^ T cells were significantly higher in the DM_R_ group as compared with unvaccinated controls (Figure 5, H and I). Overall, the frequency of IFN-γ^+^ cells increased over time in all groups.

## Discussion

T cell activation following antigen stimulation triggers dramatic changes in gene expression and function, resulting in expansion coupled with differentiation during which T cells acquire effector functions to mediate antigen clearance (23). Following antigen clearance, the process of effector T cell attrition generates a stable pool of long-lived memory T cells equipped to mount rapid recall responses upon antigen reexposure. This process of effector to memory transition constitutes the basis of immune memory following immunization or infection (24). Consequently, there is a great deal of interest in targeting pathways that augment effector to memory transition to enhance vaccine efficacy. Evidence that reducing mTOR Complex 1 (mTORC1) signaling limits attrition of CD8^+^ T cell effectors leading to an increase in the quantity and quality of memory CD8^+^ T cells (7, 9, 25), led us to determine whether fine-tuning mTORC1 activity might augment CD8^+^ T cell responses to an HIV vaccine platform. Our data show that rapamycin treatment during the MVA boosts of a DNA/MVA SIV vaccine regimen enhances the functional quality of vaccine-elicited memory CD8^+^ and CD4^+^ T cells, markedly restricting pathogenic SIV replication.

The effect of rapamycin on vaccine-elicited CD8^+^ T cells in the rhesus model was previously demonstrated in the vaccinia virus immunization setting (15). Rapamycin treatment during immunization enhanced the quality of MVA-specific CD8^+^ T cells, resulting in enhanced recall responses after vaccinia virus challenge (15). Studies elucidating cellular programs underlying enhanced functionality of CD8^+^ T cells have observed enrichment for transcripts involved in programs regulating cell survival in elite controllers — in line with our current findings of increased expression of survival proteins Bcl2 and IL-7R (26). Indeed, studies in HIV^+^ people have identified that IL-7R expression on HIV-specific CD8^+^ T cells inversely correlates with viral set point (27). In line with these data, a recent study demonstrated that generation of functional CD8^+^ T cells from exhausted CD8^+^ T cells in HIV^+^ patients ex vivo resulted in downregulation of the mTORC1 pathway and concomitant induction of IL-7R (28). Correspondingly, constitutive mTORC1 activation results in terminally differentiated CD8^+^ T cell effectors unable to transition to memory cells (29). Thus, inhibiting mTORC1 with rapamycin would be expected to promote differentiation of Tcm cells typified by expression of CCR7, IL-7R, and Bcl2, resulting in enhanced ability to mount strong recall responses.

While fine-tuning mTOR activity enhances memory differentiation in CD8^+^ T cells, some level of mTOR activation is critical for both CD4^+^ and CD8^+^ T cell effector differentiation. mTOR-null CD4^+^ T cells fail to differentiate into effectors and are instead diverted to Foxp3^+^ Tregs, in part, due to heightened basal TGF-β signaling (5), and treatment with rapamycin furthermore expands Tregs ex vivo (30). In the present study, we did not observe increased generation of CD4^+^ Tregs following rapamycin treatment (data not shown); it should be noted, however, that skewing to regulatory CD4^+^ subsets is dependent on the dose and duration of rapamycin, and it is likely that the threshold of mTOR inhibition for CD4^+^ T cell polarization to Tregs was not achieved in the current studies (31). We did, however, observe that rapamycin treatment induced memory CD4^+^ T cells with reduced reexpression of CCR5 during recall proliferation ex vivo. The expression of CXCR3 was similar (data not shown), suggesting CD4 Th1 differentiation induced by our vaccine regimen in the presence of rapamycin (32) was not impaired. This observation implies that specific effects on the CCR5 locus such as epigenetic changes might be at play (17). In line with our observations, transcriptomic analysis of Env-stimulated peripheral T cells in RV144 vaccine recipients versus placebo controls at 2 weeks after final immunization identified activation of the mTORC1 pathway with risk of acquisition, invoking a role for an mTOR-mediated increase in CCR5^+^ target cells as a putative mechanism (33). This hypothesis is supported by data showing that rapamycin causes downregulation of CCR5 on CD4^+^ T cells in vitro (17). Furthermore, rapamycin treatment in ART-suppressed macaques chronically infected with SIV239 resulted in downregulation of CCR5 transcripts in peripheral blood and inhibited CD4^+^ T cell proliferation (8). All together, our findings of reduced CCR5 reexpression are in concordance with the literature (17), suggesting the potential for optimized mTORC1 inhibition as an approach to reduce generation of target CD4^+^ T cells.

We also observed that inhibiting mTOR during both the DNA prime and MVA boosts (D_R_M_R_) negatively influenced the magnitude of SIV-specific IFN-γ^+^CD4^+^ T cell responses. Since CD4^+^ T cells are effectively induced following the DNA prime (34, 35), our data indicate that effective expansion of vaccine-specific CD4^+^ T cell responses was attenuated by rapamycin treatment. These data emphasize that, in the context of a prime-boost vaccine regimen, selective inhibition of mTORC1 during the boost may be more effective in preserving magnitude while the enhancing functional quality of vaccine-elicited T cells.

In summary, our findings add to our understanding of mTORC1 signaling in vaccination. We report that rapamycin treatment during vaccination enhanced the magnitude and quality of postinfection CD8^+^ T cell responses by enhancing the development of CD8^+^ Tcm cells. We furthermore demonstrate that rapamycin treatment decreased CCR5 reexpression on antigen-specific CD4^+^ T cells during recall, likely contributing to enhanced CD4^+^ T cell preservation after infection. Our findings suggest that strategic inhibition of mTORC1 may represent an approach to strengthen vaccine-induced memory CD8^+^ T cell responses to mediate potent viral control.

## Methods

### Sex as a biological variable.

Only male rhesus macaques were included in the study. This study was conducted between 2011 through the end of 2012, when sex as a biological variable was not a required consideration.

### DNA and MVA vaccines.

The DNA/MVA vaccines are as described in previous studies (36). Briefly, in the DNA/SIV vaccine, the SIVmac239 Gag, Pr, RT, Env, Tat, and Rev genes are expressed by a single CMV intermediate-early promoter with intron A and the subgenomic splicing of the mRNA. A single recombinant MVA expresses SIVmac239 Gag, Pr, RT, and Env (37). The DNA/SIV vaccine expresses the complete gp160 form of Env and the MVA/SIV vaccine encodes a gp150 form of Env that has a truncation of 146 amino acids at the C-terminus of its gp41 subunit for stability and enhanced expression on the plasma membrane of infected cells (38).

### Animals, immunizations, and challenges.

All male Indian-origin rhesus monkeys (*Macaca* *mulatta*) utilized in the study were housed at the Emory National Primate Research Center.

Macaques were typed for the Mamu-A*01, Mamu-B*08, and Mamu-B*17 alleles, as described previously (Supplemental Table 1) (39–41). *TRIM5* genotype was determined by sequence analysis of PCR fragments representing the *TRIM5 TFP*, *CYPA*, and *Q* alleles, as described previously (42). Mamu-A*01, Mamu-B*08, Mamu-B*17, and *TRIM5* genotypes were distributed evenly across the vaccination groups. In each group, 4 animals were Mamu-A*01 positive, 1 animal was Mamu-B*08 positive, and 1 animal was Mamu-B*17 positive.

Three groups of 10 macaques each were primed with DNA/SIV on weeks 0 and 8 and boosted with MVA/SIV vaccine on weeks 16 and 24. One group (D_R_M_R_) was treated with rapamycin during prime and boost, the second group (DM_R_) received rapamycin treatment only during boost, and the third group (DM) was not treated with rapamycin, and served as the vaccinated, untreated control. Another group of 10 macaques did not receive any vaccine and served as unvaccinated controls.

Weekly intrarectal challenges were administered starting 21–24 weeks after the final MVA immunization using 647 TCID_50_ (1.25 × 10^7^ copies of viral RNA) of SIVmac251 (stock made by Ron Desrosiers, day 9 harvest, dated 02/18/2006). Single-genome amplification analysis in 4 unvaccinated monkeys demonstrated 1–2 transmitted virus variants/animal. A total of 8 challenges were given, one extra challenge after all the controls were infected after the seventh challenge.

### Rapamycin preparation and administration.

Rapamycin (LC Laboratories, R-5000) was prepared at 2 mg/mL by dissolving in 80% PEG (MW = 400, Sigma-Aldrich, P3265), 10% Tween 80 (Sigma-Aldrich, P6474), and 10% *N*,*N*-dimethylacetamide (Sigma-Aldrich, D137510). The resulting solution was filter sterilized, aliquoted, and stored at –80°C until further use. Daily administration of rapamycin (5–50 μg/kg/day) was given intramuscularly starting from 3 days before each DNA or MVA vaccination. The treatment was started with 50 ug/kg/day loading dose and the dose was decreased on a weekly basis based on plasma concentration of rapamycin to achieve a target concentration of 5–15 ng/mL of plasma.

### Collection and processing of blood, rectal biopsies, and secretions.

PBMCs were isolated from whole blood using cell preparation tubes according to the standard procedures, as described previously (43). Lymphocytes from 10–20 pinch rectal biopsies were obtained as described previously (43). Rectal secretions were collected and eluted from Weck-Cel sponges as previously described (43).

### T cell responses.

The frequency of Gag-specific CD8^+^ T cells was enumerated using tetramer staining. PBMCs isolated from fresh blood collected in cell preparation tubes were stained with a viability dye (Fixable Viability Dye, eBioscience) in 1× PBS followed by staining with a cocktail containing antibodies against CD4 (clone OKT4, BioLegend), CD28 (clone CD28.2, BioLegend), CD127 (clone eBioRDR5, eBioscience), CCR7 (clone G043H7, BioLegend), CXCR3 (clone G025H7, BioLegend), CD95 (clone DX2, BioLegend), CD45Ra (clone MEM-56, Invitrogen), CD8a (clone RPA-T8, BioLegend), and CD3 (clone SP34-2, BD Biosciences), and the Mamu-A*01 MHC-I tetramer specific for SIVmac239 Gag immunodominant peptide CM9 (181-CTPYDINQM-189) conjugated to allophycocyanin in PBS containing 5% FBS (Corning Life Sciences). Cells were then fixed and permeabilized using the Fix/Perm and Perm/Wash buffers (BD Biosciences) before intracellular staining for Ki67 (clone B56, BD Biosciences), Bcl2 (clone Bcl2/100, BD Biosciences), and granzyme B (clone GB12, Invitrogen). The samples were analyzed using a LSR-II flow cytometer (BD Biosciences).

Functional responses of Gag- and Env-specific CD8^+^ and CD4^+^ T cells were measured using an ICS assay as described previously (43). One to 2 million PBMCs were incubated in 200 μL of RPMI 1640 medium containing 10% FBS with anti-CD28 (1 μg/mL; clone CD28.2, BD Biosciences), anti-CD49d (1 μg/mL; clone 9F10, BD Biosciences), and different stimulation conditions as follows: (a) DMSO as a negative control; (b) Gag peptide pool (1–125 peptides derived from SIVmac239; catalog no. 6204, NIH AIDS reagent program), consensus subtype-A Env peptide pool (1–110 peptides that induced detectable T cell responses consistently; catalog no. 12734, NIH AIDS reagent program) at a concentration of 1 μg/mL; and (c) phorbol myristate acetate and ionomycin for positive control. Anti–human CD107a (clone H4A3, BioLegend) was added to the culture. Brefeldin A (10 μg/mL) was added after 2 hours of incubation and cells were incubated for an additional 4 hours. Cells were transferred to 4°C overnight and stained the next day for expression of cytokines. Cells were washed once with PBS and stained with a viability dye followed by staining with a surface antibody cocktail containing antibodies against CD8 and CD4 (clones same as above). Cells were then washed, fixed, and permeabilized with Cytofix/Cytoperm buffer for 10 minutes. Permeabilized cells were stained with ICS antibodies against IFN-γ (clone 4S.B3, BioLegend), TNF-α (clone Mab11, eBioscience), IL-2 (clone MQ1-17H12, BioLegend), IL-4 (clone 8D4-8, BioLegend), CD40L (clone 24-31, BioLegend), and CD3. Cells were then washed twice with Perm/Wash buffer and once with staining buffer before analysis using a BD LSR-II flow cytometer. All flow cytometry data were analyzed using FlowJo software v.10 (TreeStar).

The CFSE staining was performed as described previously (44). Briefly, PBMCs were prestained with CFSE, and 1 × 10^6^ cells were stimulated in 48-well plates in a volume of 600 μL in RPMI 1640 containing 10% human serum at 37°C under 5% CO_2_. Cells were stimulated with pooled peptides spanning the entire SIV Gag protein (single pool of 125 peptides, catalog no. 6204, NIH AIDS Research and Reference Reagent Program) at a concentration of 1.2 μg/mL of each peptide. Unstimulated cells served as negative controls. After 5 days in culture, ICS was performed using antibodies specific for CD3 (clone SP34-2, BD Biosciences), CD4 (clone L200, BD Biosciences), CD8 (clone SK1, BD Biosciences), CCR5 (clone 3A9, BD Biosciences), and Ki-67 (clone B56, BD Biosciences). Flow cytometry data were analyzed using FlowJo software.

### Quantitation of SIV RNA plasma load.

The SIV copy number was determined using quantitative real-time PCR, as previously described (45). RNA from all specimens was extracted and amplified in duplicate, with the mean results reported. For viral load determinations in gut, total RNA was extracted from approximately 1 million cells obtained from gut biopsies and used for quantitative real-time PCR analyses.

### Statistics.

Statistical analyses were conducted using Prism (GraphPad Software). The 1-way ANOVA test was used to compare immune responses and viral RNA levels between groups, with the nonparametric Kruskal-Wallis test being used for groups of more than 4 animals. In some cases the unpaired, nonparametric Mann-Whitney test was used to compare immune responses between groups, this is annotated in the figures and in the figure legends. Spearman’s rank correlation method was used for nonparametric data correlations (indicated as *r* values). A 2-sided *P* value of less than 0.05 was considered significant. The log-rank (Mantel-Cox) test was used for the survival and acquisition curves. The box and whisker plots show the maximum value (the highest data point) and the bottom of the whisker (the lowest data point). It also includes all of the data points.

### Study approval.

All procedures involving animals were performed under the standards of the NIH *Guide for the Care and Use of Laboratory Animals* (National Academies Press, 2011) and protocols approved by the Emory University (Atlanta, Georgia, USA) Institutional Animal Care and Use Committee (IACUC) (protocol 092-2010Y). The Emory National Primate Center is accredited by American Association for Accreditation of Laboratory Animal Care (AAALAC).

### Data availability.

The data are available in the Supporting Data Values file or from the corresponding author upon request.

## Author contributions

SS conducted experiments, acquired and analyzed data, and contributed to the writing of the manuscript. KS analyzed data and contributed to the writing of the manuscript. SK conducted experiments, acquired and analyzed data. RB conducted experiments. SG provided reagents and conducted experiments. RA contributed to the study design and manuscript writing. SSI conducted experiments, acquired and analyzed data, and contributed to the writing of the manuscript. KA contributed to the study design. RRA acquired funding, and contributed to the study design and manuscript writing.

## Conflict of interest

The authors have declared that no conflict of interest exists.

## Funding support

This work is the result of NIH funding, in whole or in part, and is subject to the NIH Public Access Policy. Through acceptance of this federal funding, the NIH has been given the right to make the work publicly available in PubMed Central.

- NIH/NIAID grants R01 AI057029, R01 AI071852, and R01 AI074417 to (RRA).
- Emory National Primate Research Center base grant P51 RR00165.
- Emory CFAR grant P30 AI050409.
- NIH Office of Research Infrastructure Programs OD grant P51OD011132 to the Emory Primate Center.

## Supplementary Material

Supplemental data

Supporting data values

## Version 1 04/23/2026

In-Press Preview

## Version 2 06/08/2026

Electronic publication
