# Supplementary material for: Coadministration of rapamycin with a DNA/MVA SIV vaccine improves memory CD8+ T cell response
Source: JCI Insight. 2026 Apr 23;11(11):e193752. doi: 10.1172/jci.insight.193752 (PMC13313493; doi:10.1172/jci.insight.193752)
Supplement: Supplemental data [file jciinsight-11-193752-s198.pdf]

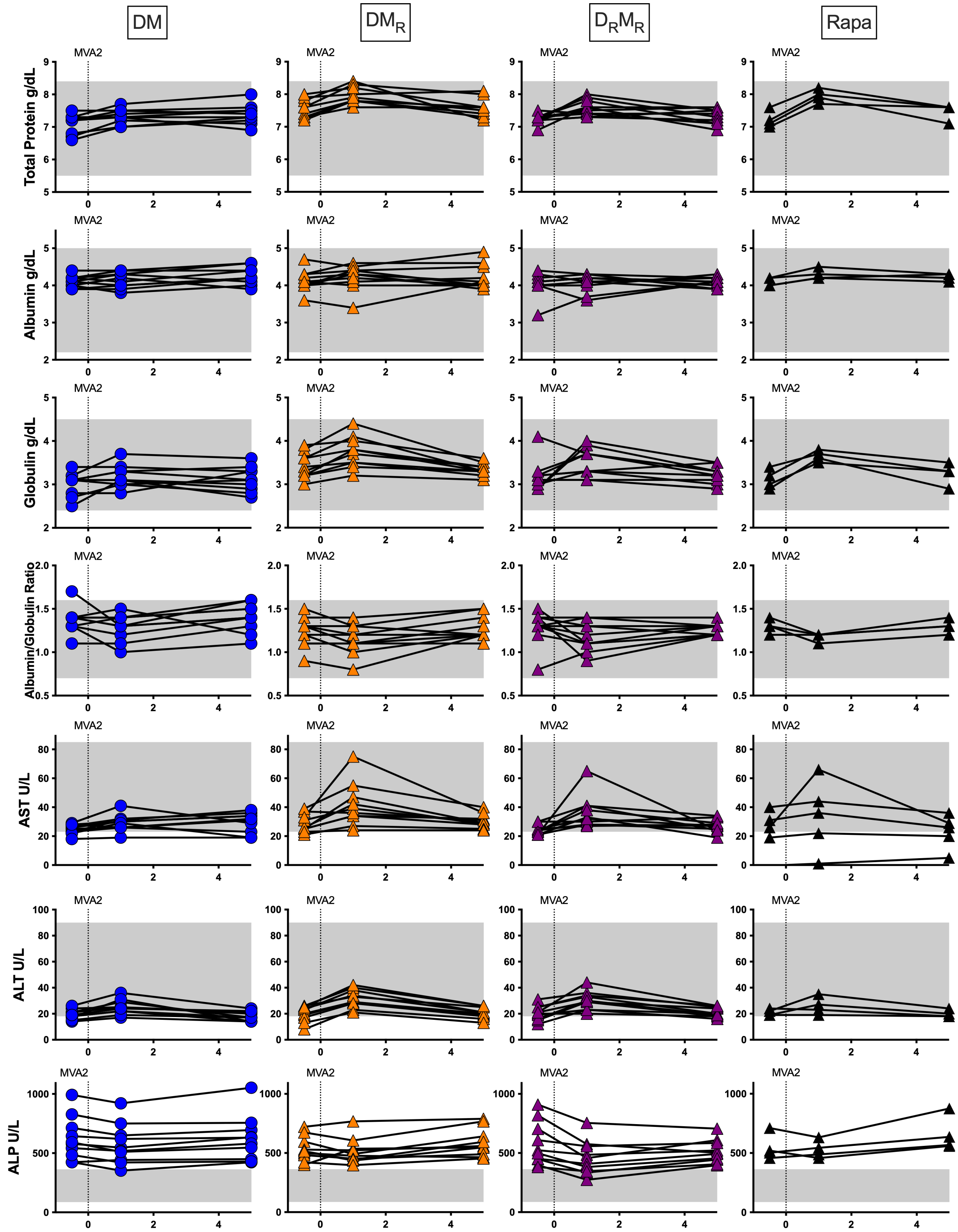

DM
DM$_R$
D$_R$M$_R$
Rapa
MVA2
Total Protein g/dL
Albumin g/dL
Globulin g/dL
Albumin/Globulin Ratio
AST U/L
ALT U/L
ALP U/L

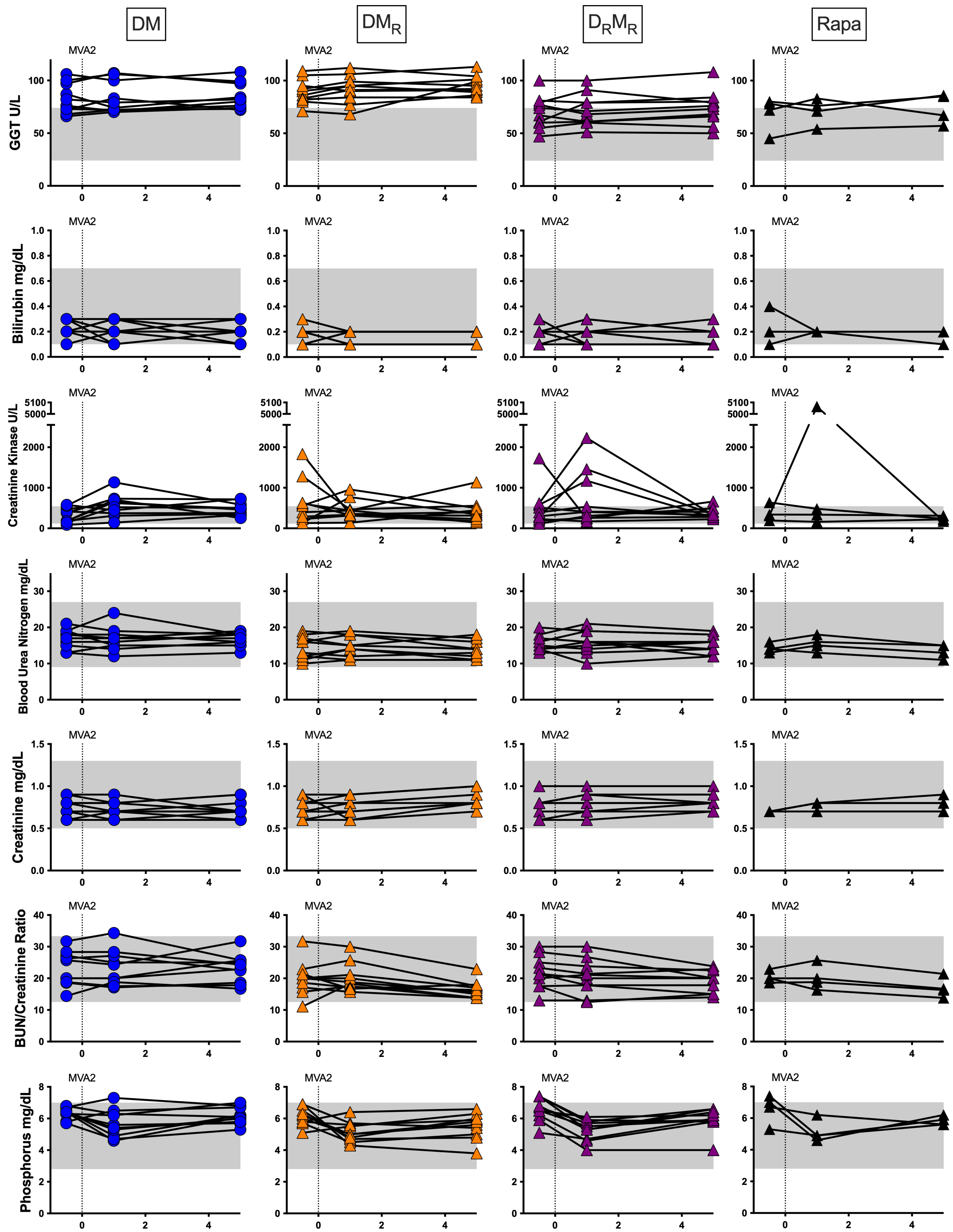

DM
DM$_R$
D$_R$M$_R$
Rapa
MVA2
GGT U/L
Bilirubin mg/dL
Creatinine Kinase U/L
Blood Urea Nitrogen mg/dL
Creatinine mg/dL
BUN/Creatinine Ratio
Phosphorus mg/dL

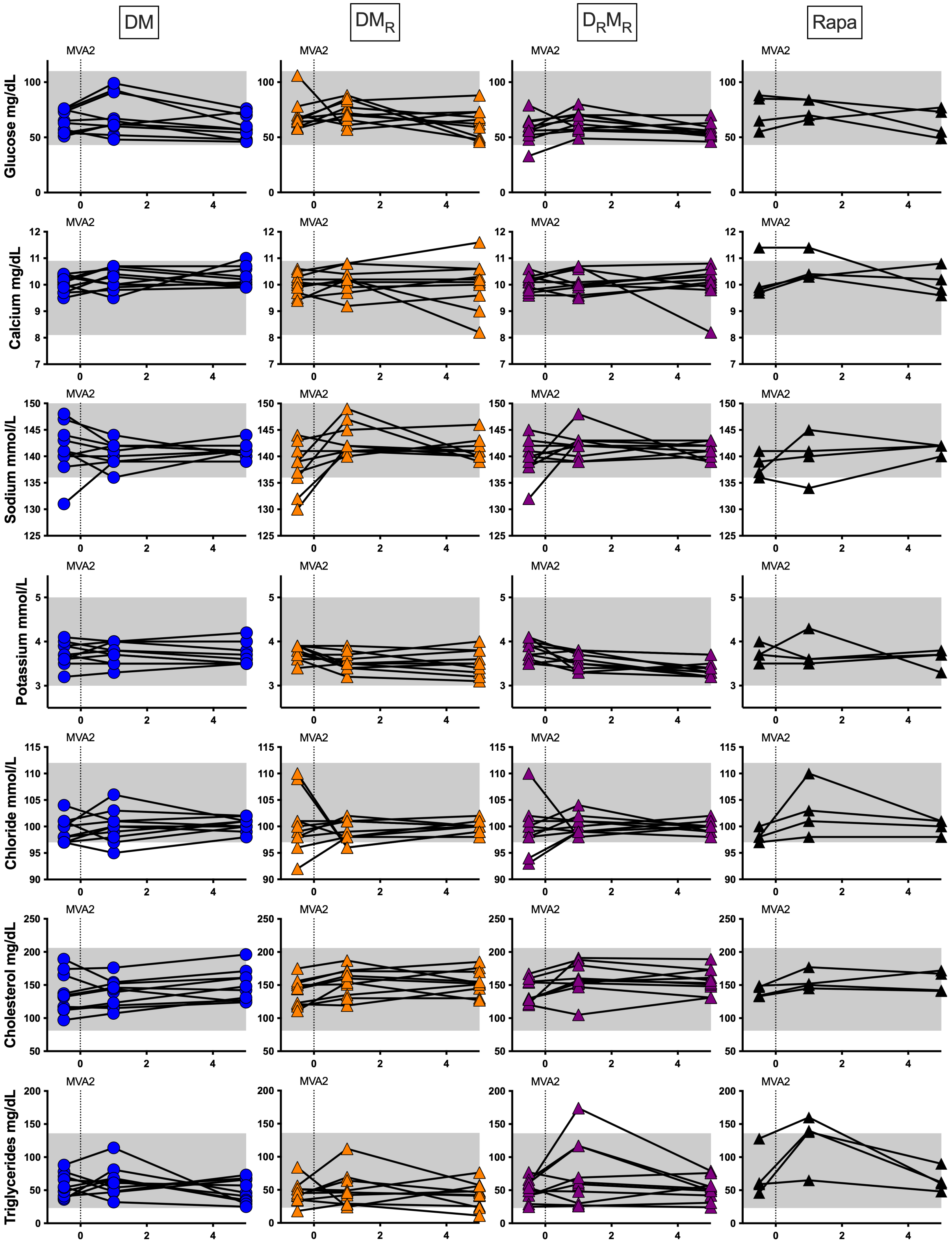

DM
$DM_R$
$D_RM_R$
Rapa
MVA2
Glucose mg/dL
Calcium mg/dL
Sodium mmol/L
Potassium mmol/L
Chloride mmol/L
Cholesterol mg/dL
Triglycerides mg/dL

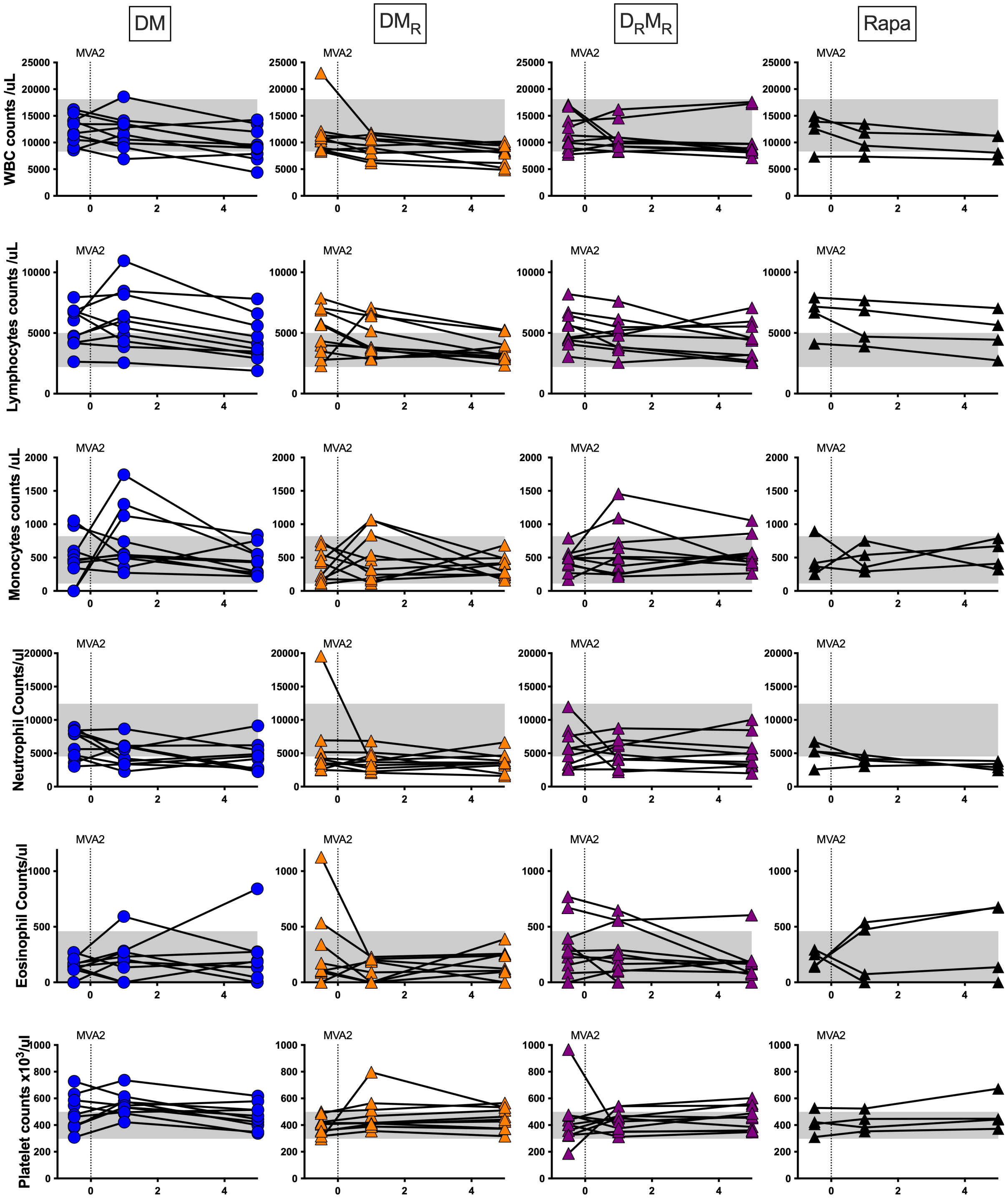

DM
DMR
DRMR
Rapa
MVA2
WBC counts /uL
Lymphocytes counts /uL
Monocytes counts /uL
Neutrophil Counts/ul
Eosinophil Counts/ul
Platelet counts x10³/ul

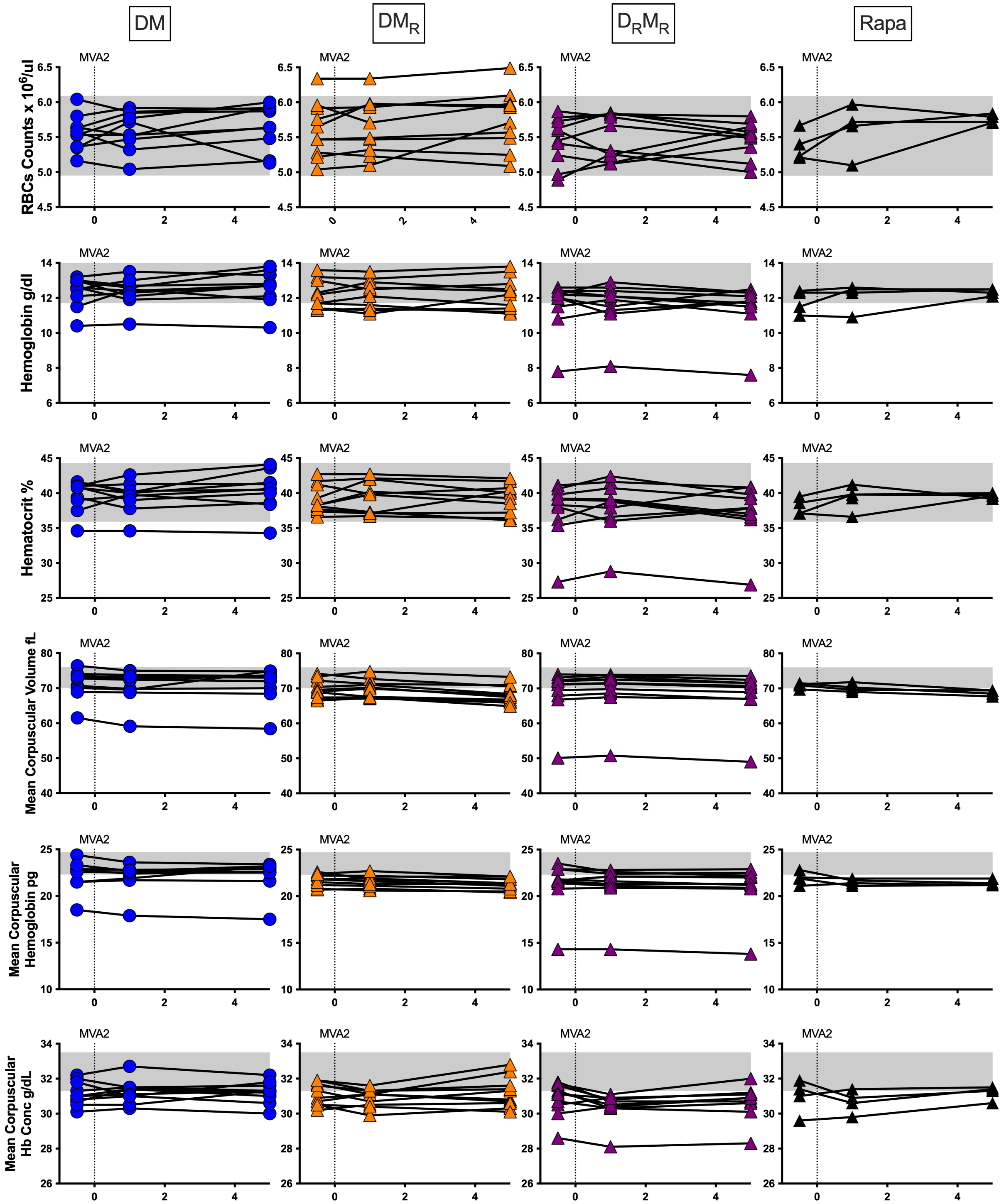


**Figure S1. Rapamycin treatment does not alter blood chemistry or hematology profiles.**

Blood chemistry, liver enzymes, kidney enzymes, and hematology following the second MVA.

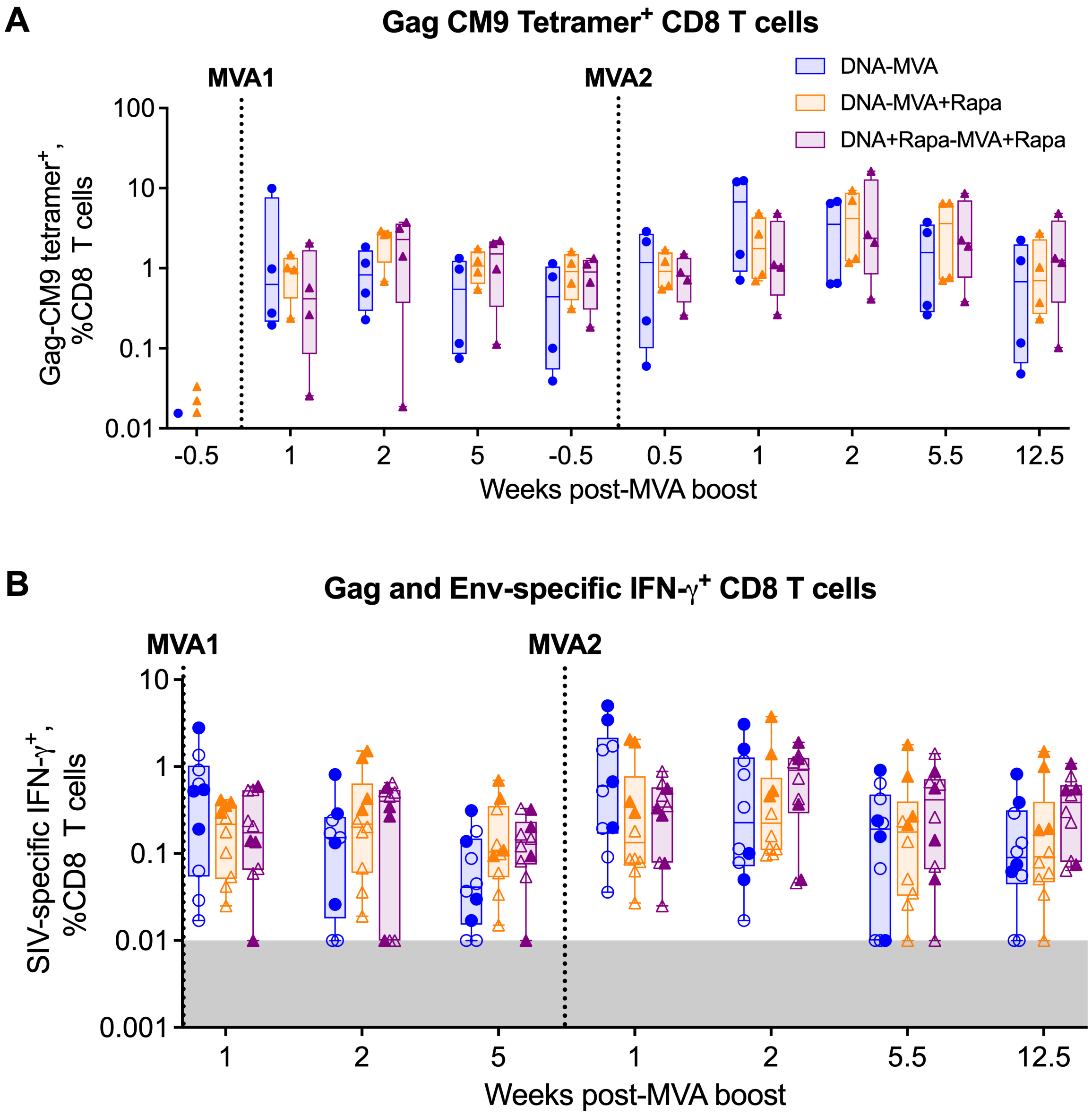


**Figure S2. Rapamycin treatment delays peak and reduces Gag- and Env- specific tetramer cells. A.** Frequency of Gag-CM9 tetramer$^{+}$ CD8 T cells shown individually after MVA vaccinations (n = 4/group). **B.** Frequency of SIV-specific IFN-γ$^{+}$ CD8 T cells shown individually after MVA vaccinations (n = 10/group).

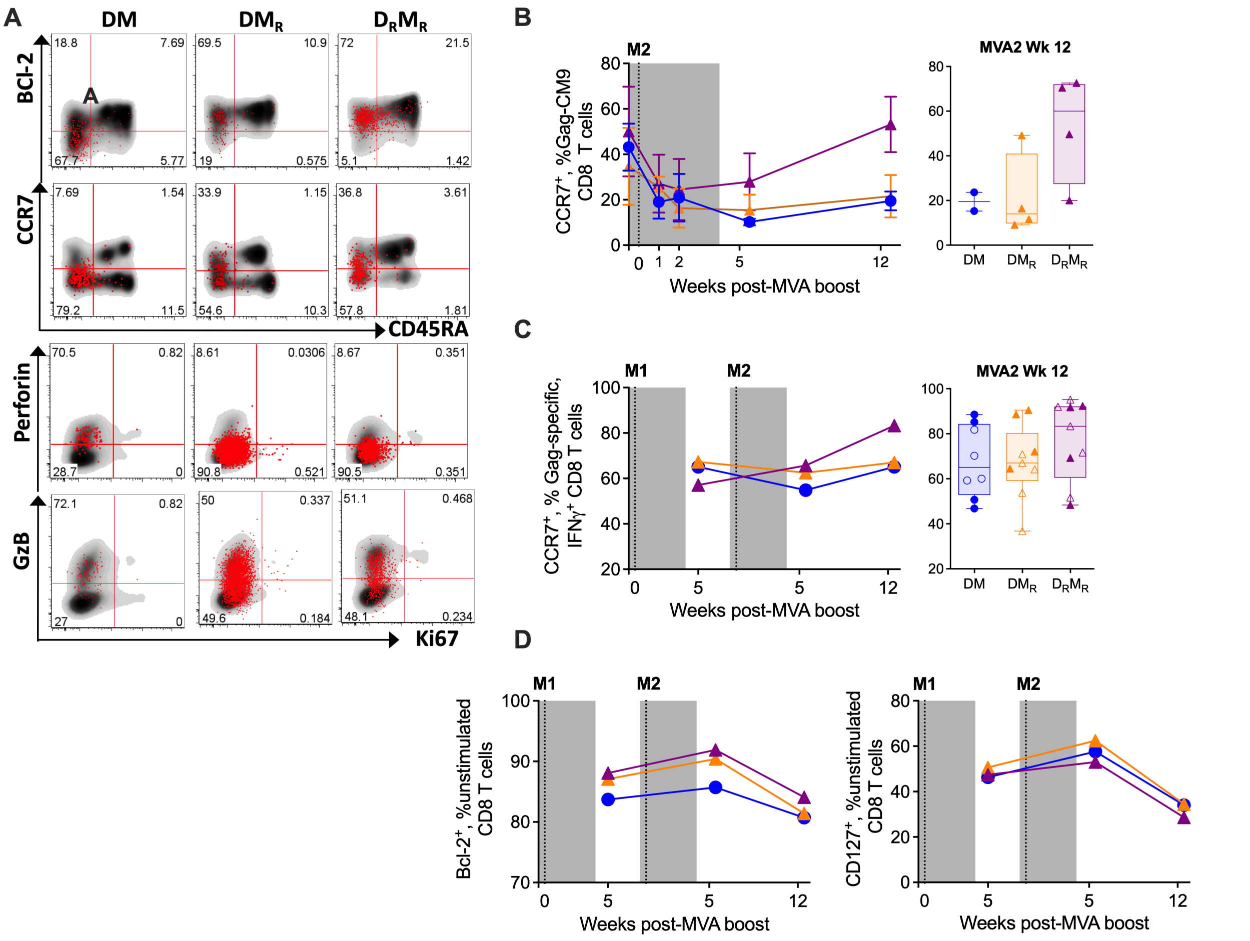


**Figure S3. Rapamycin specifically enhances Bcl-2 and CD127 expression in vaccine-induced CD8 T cells. A.** Representative flow plots of Bcl-2 and CCR7 against CD45RA, and Perforin and GzB against Ki67 on total CD8 T cells (black and gray density plot) and tetramer$^+$ CD8 T cells (red dots). **B.** Geometric means of CCR7$^+$ frequencies on Gag-CM9 specific tetramer+ CD8 T cells measured temporally post-MVA2. Individual data at MVA2 week 12 (n = 4/group). **C.** Geometric means of CCR7$^+$ frequencies on Gag-specific IFN-γ$^+$ CD8 T cells measured temporally post-MVA vaccinations. Individual data at MVA2 week 12 (n = 10/group). **D.** Geometric means of Bcl-2$^+$ and CD127$^+$ frequencies on total CD8 T cells measured temporally post-MVA vaccinations. Statistics: Unpaired, non-parametric Mann-Whitney tests were used to compare marker$^+$ frequencies between groups.

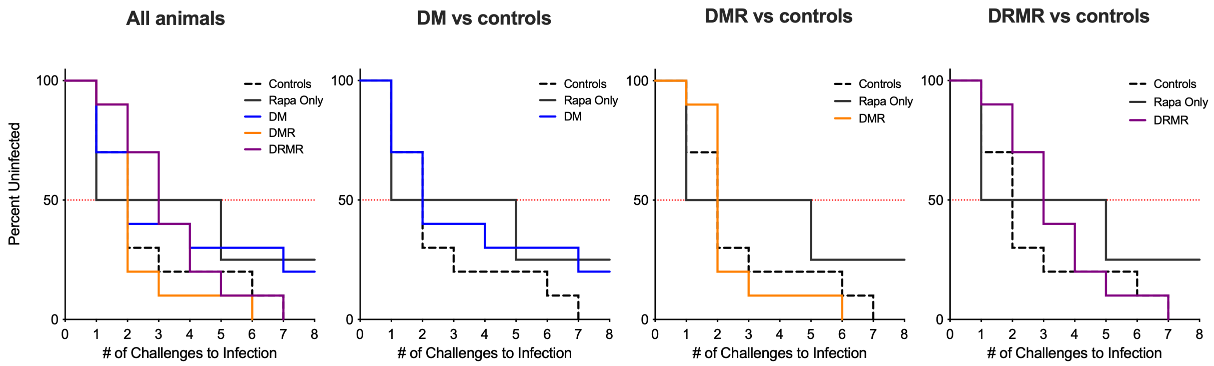


**Figure S4. Kaplan-Meier Acquisition Curve.** Infection curve for all groups. Statistics: Log-rank (Mantel-Cox) test.

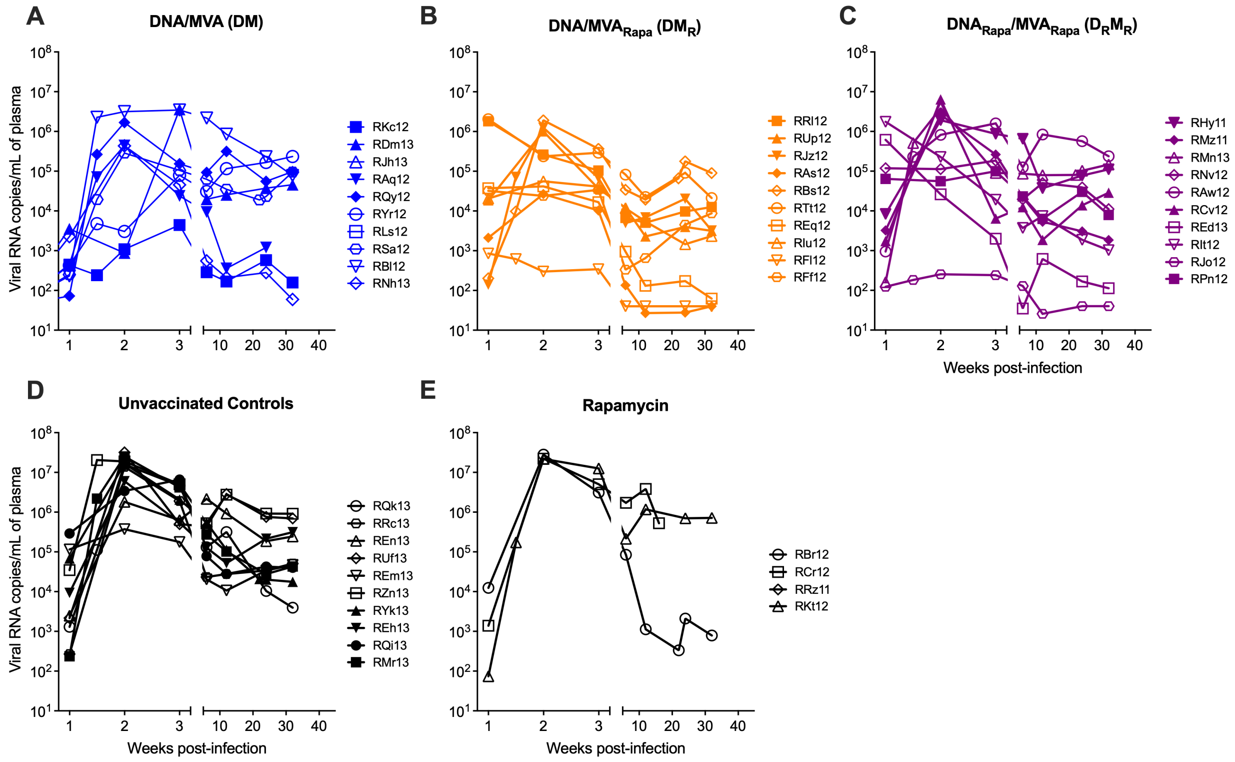


**Figure S5. Viral load data of individuals animals across all experimental groups. A–E.** Plasma viral load data for individual macaques belonging to groups in the following order: DM, $DM_R$ $D_RM_R$, UC, and Rapa.

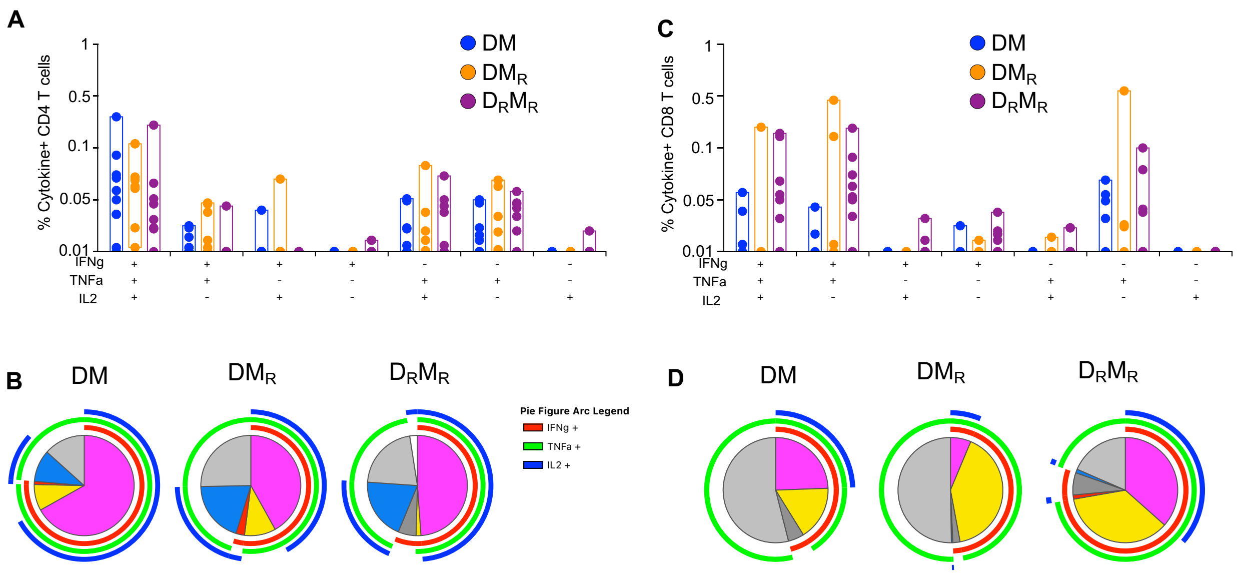


**Figure S6. Polyfunctionality of Gag-specific CD4 and CD8 T cells at week 12 post 2nd MVA boost in blood of vaccinated animals. (A, C)** The magnitude of triple, double and single cytokine+ cells as a percent of total (A) CD4 or (C) CD8 T cells. Each dot represents an individual monkey. **(B, D)** The relative distribution of triple, double and single cytokine+ cells as a percent of total cytokine+ (B) CD4 or (D) CD8 T cells. For the pie graphs, the mean value for each subset for all animals in a group is plotted.

| | red A01+ | | Macaque Genotypes | | | | | | | Macaque Genotypes | | |
|---|---|---|---|---|---|---|---|---|---|---|---|---|
| **Group** | **Monkey** | **Challenge # infected** | **A*01** | **B*08** | **B*17** | | **Group** | **Monkey** | **Challenge # infected** | **A*01** | **B*08** | **B*17** |
| DM | RKc12 | **2** | + | - | - | | DRMR | RHy11 | **7** | + | - | - |
| DM | RDm13 | **2** | + | - | - | | DRMR | RMz11 | **5** | + | - | - |
| DM | RJh13 | **UI** | - | + | - | | DRMR | RMn13 | **1** | - | - | - |
| DM | RAq12 | **4** | + | - | - | | DRMR | RNv12 | **4** | - | - | - |
| DM | RQy12 | **1** | + | - | - | | DRMR | RAw12 | **4** | - | - | - |
| DM | RYr12 | **7** | - | - | - | | DRMR | RPn12 | **3** | + | - | - |
| DM | RLs12 | **UI** | - | - | + | | DRMR | RCv12 | **2** | + | - | - |
| DM | RSa12 | **1** | - | - | - | | DRMR | REd13 | **2** | - | + | - |
| DM | RBl12 | **1** | - | - | - | | DRMR | RIt12 | **3** | - | - | - |
| DM | RNh13 | **2** | - | - | - | | DRMR | RJo12 | **3** | - | - | + |
| | | | | | | | | | | | | |
| DMR | RRl12 | **2** | + | - | - | | UC | RQk13 | **1** | - | - | - |
| DMR | RUp12 | **2** | + | - | - | | UC | RRc13 | **2** | - | - | - |
| DMR | RJz12 | **2** | + | - | - | | UC | REn13 | **2** | - | - | - |
| DMR | RAs12 | **3** | + | - | - | | UC | RUf13 | **2** | - | - | - |
| DMR | RBs12 | **1** | - | - | - | | UC | REm13 | **7** | - | - | - |
| DMR | RTt12 | **2** | - | - | - | | UC | RZn13 | **6** | - | - | - |
| DMR | REq12 | **2** | - | - | - | | UC | RYk13 | **1** | + | - | - |
| DMR | RIu12 | **6** | - | + | - | | UC | REh13 | **2** | + | - | - |
| DMR | RFl12 | **2** | - | - | + | | UC | RQi13 | **3** | + | - | - |
| DMR | RFf12 | **2** | - | - | - | | UC | RMr13 | **1** | + | - | - |
| | | | | | | | | | | | | |
| | | | | | | | R | RBr12 | **1** | - | + | - |
| | | | | | | | R | RCr12 | **1** | - | - | - |
| | | | | | | | R | RRz11 | **UI** | - | - | - |
| | | | | | | | R | RKt12 | **5** | - | - | - |

**Table S1.** The Mamu typing information for study animals along with the challenge number at which an animal was productively infected. UI, uninfected.
